# Semaglutide Prevents Aortic Rupture and Dissection in the Angiotensin II Mouse Model

**DOI:** 10.3390/biomedicines14040933

**Published:** 2026-04-20

**Authors:** Amanda Balboa Ramilo, Kevin Mani, Anders Wanhainen, Henrik Lodén, Anna Nilsson, Per E. Andrén, Malou Friederich-Persson, Dick Wågsäter

**Affiliations:** 1 Department of Medical Cell Biology, Uppsala University, 751 23 Uppsala, Swedenmalou.friederich@mcb.uu.se (M.F.-P.); 2 Department of Surgical Sciences, Uppsala University, 751 85 Uppsala, Sweden; kevin.mani@uu.se (K.M.); anders.wanhainen@uu.se (A.W.); 3 Department of Pharmaceutical Biosciences, Spatial Mass Spectrometry, Science for Life Laboratory, Uppsala University, 751 23 Uppsala, Sweden; henrik.loden@uu.se (H.L.); per.andren@uu.se (P.E.A.)

**Keywords:** abdominal aortic aneurysm, semaglutide, rupture, dissection

## Abstract

**Background and aims:** Abdominal aortic aneurysm (AAA) is a vascular disease characterized by the progressive dilation of the aorta, culminating in rupture. At present, there are no pharmacological treatments to prevent AAA development or reduce rupture rate. A recent study showed that patients prescribed Glucagon-like peptide-1 receptor agonists (GLP-1RAs) have significantly lower risks of mortality, AAA repair, and acute abdominal aortic syndrome. Semaglutide is a GLP-1RA with increased agonist capacity and longer half-life, compared to earlier generations of GLP-1RAs. In this study, we aimed to investigate the role and mechanisms of semaglutide in the prevention of AAA development and rupture in a murine model. **Methods:** AAA was induced in apolipoprotein-E-deficient mice, by continuous subcutaneous infusion of angiotensin II. Treatment with semaglutide (12 µg/kg) began seven days after disease induction (rescue trial) or simultaneously with disease induction (prophylactic trial). At experimental endpoint, aortic diameter was measured by high-frequency ultrasound and the aortic tissue was collected for histological analysis. **Results:** Prophylactic treatment with semaglutide drastically reduced mortality by dissection and rupture during the first seven days of disease development, but did not affect AAA formation at 28 days. Histological evaluation of the aorta at day seven showed a normal vessel wall thickness with a trend for a higher content of collagen in the aortic wall in mice treated with semaglutide, compared to controls. **Conclusions:** Semaglutide prevents aortic rupture and dissection in the early phases of AAA development in the angiotensin II mouse model, likely by promoting the maintenance of an adequate proportion of collagen in the vessel wall.

## 1. Introduction

An abdominal aortic aneurysm (AAA) is defined as a dilation of the aortic lumen to a diameter > 30 mm. It affects >5% of male smokers over the age of 65 and less than 1% of non-smokers [1]. Widening of the aortic lumen is caused by progressive degeneration of the aortic wall due to vascular smooth muscle cell (VSMC) loss and degradation of the extracellular matrix (ECM). Elastin and collagen are two of the main components of the ECM. Loss of elastin is associated with progressive dilation of the vessel, while collagen loss leads to rupture. Ruptured aneurysms have a mortality rate of 65–80% [2].

No pharmacological treatment exists to prevent or reduce AAA development or rupture. Metformin, a commonly used treatment for type 2 diabetes mellitus (T2DM), is the only drug with promising effects [3], with clinical trials ongoing [4].

Glucagon-like peptide-1 receptor agonists (GLP-1RAs) are a class of drugs used in the management of T2DM [5], exerting their action through the glucagon-like peptide-1 receptor (GLP-1R). Although the main effect of GLP-1Ras is antidiabetic, promoting insulin release in a glucose-dependent manner, they also have cardiovascular protective effects [6]. In the vasculature, GLP-1R activation results in dilation of microvessels and modulation of mitochondrial metabolism in VSMCs [6], which is a recognized key aspect in clinical and preclinical AAA progression [7].

GLP-1RAs have been proven efficient in the treatment of AAA in rodent models, mainly by preserving the ECM and reducing macrophage infiltration [8]. Under the preparation of the current manuscript, Ahn and colleagues showed that patients prescribed GLP-1RAs have significantly lower risks of mortality, AAA repair, and acute abdominal aortic syndrome in both T2D (OR 0.56, 95% CI 0.47–0.66) and non-T2D cohorts (OR 0.42, 0.28–0.63) [9]. Semaglutide is a newer GLP-1RA with an increased agonist capacity and longer half-life, compared to other drugs in this class [5]. It efficiently prevents atherosclerosis development in apolipoprotein E knockout (ApoE KO) mice [10] and affects vascular remodeling by normalizing collagen content in cardiac fibroblasts [11] and the aortic wall [12]. An atherosclerotic background in mice, such as ApoE KO, is widely used because they develop aneurysms similar to human pathophysiology [7]. Despite its increased agonist capacity, the effects of semaglutide on AAA initiation and progression has not yet been explored. In this study, we aimed to investigate the role and mechanisms of semaglutide in initiation of AAA (with a prophylactic-style trial) and in prevention of AAA progression (with a rescue-style trial).

## 2. Materials and Methods

### 2.1. AAA Model

All animal experiments were approved by the Uppsala region animal ethics committee board (5.8.18-10005/2021 and 5.8.18-15489/2022).

AAA was induced in male ApoE-deficient mice, 8–10 weeks old, by continuous subcutaneous infusion of angiotensin II (angII, 1000 ng/kg/min), from a mini-osmotic pump, model 1004 (Alzet, Cupertino, CA, USA). The control group received saline solution. At experimental endpoint, aortic diameter was measured by high-frequency ultrasound (Vevo 11000, Fujifilm Visualsonics Inc., Toronto, ON, Canada). AAA was defined as the presence of an aortic diameter superior to 1.5 mm or death by dissection/rupture.

**Figure 1:**
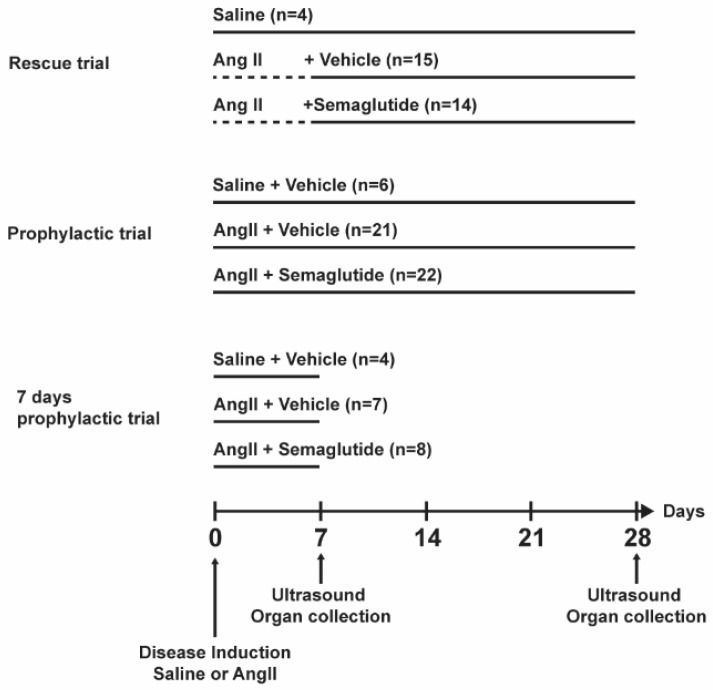
Representation of the experimental timeline for the rescue, prophylactic and seven-day prophylactic trials. At day 0, a mini-osmotic pump was implanted subcutaneous in all mice, with saline or angiotensin II (angII). In the rescue trial, treatment with vehicle or semaglutide started at day seven for the angII-infused mice. In the prophylactic and seven-day prophylactic trial, saline or angII infusion started simultaneously with vehicle or semaglutide treatment. Mice were terminated at day 28 in the rescue and prophylactic trials and at day 7 in the seven days prophylactic trial.

Semaglutide (12 µg/kg, Cayman Chemical, MI, USA) was administered daily by subcutaneous injection. As control, the vehicle solution (5% DMSO) was used. Treatment groups were mixed in the same cages to minimize confounding factors. Three different treatment schemes were tested, as represented in Figure 1, hereon named rescue trial, prophylactic trial and seven-day prophylactic trial. In the rescue trial, treatment with semaglutide or vehicle started seven days after disease induction and continued to day 28. In the prophylactic trial, treatment started simultaneously with disease induction and continued for 28 days. In the seven-day prophylactic trial, treatment started simultaneously with disease induction and continued for seven days.

Mice were treated with buprenorphine (0.1 mg/kg) twice daily for 48 h postoperatively and mice were observed daily for any signs of pain or disability. If mice showed discomfort, pain or weight loss according to the local assessment for humane endpoint, the mice were sacrificed. In this study, no animals needed to be sacrificed due to reaching a humane endpoint.

### 2.2. Histology

Mouse aortic cryosections (10 µm) were stained by hematoxylin–eosin for luminal diameter and aortic wall thickness measurement. Evaluation of the collagen content of the aortic wall was performed with Masson’s trichrome and picrosirius red stainings. Elastin content was evaluated by Verhoeff–Van Gieson staining. Immunohistochemical detection of GLP-1R was performed with rabbit anti-GLP1-1R antibody (Bs-1559R, Bioss, Woburn, MA, USA), diluted 1:200 and incubated overnight at 4 °C. Goat anti-rabbit IgG 1:1000 (Cat no 5220-0336, SeraCare, Milfrod, MA, USA) was used as secondary antibody. Omitting the secondary antibody served as a negative control for the antibody staining. An average vessel wall area was calculated from the inner luminal diameter and outer adventitia diameter. All histological analyses were performed in a blinded manner and quantification was performed with QuPath software (version 0.7.0) and ImageJ (version 1.54r).

### 2.3. Gene Expression

Total RNA was isolated from suprarenal aortic mouse tissue with RNeasy Mini kit (Cat no 74104, Qiagen, Hilden, Germany) and treated with RNase free DNase (Cat no 79254, Qiagen, Hilden, Germany). cDNA was prepared with the SuperScript III First Strand Synthesis Super Mix (Cat no 11752-050, Invitrogen, Waltham, MA, USA) and used for quantitative polymerase chain reaction (qPCR). Gene amplification for *GLP-1R*, matrix metalloproteinase 9 (*MMP9*), matrix metalloproteinase 2 (*MMP2*), α-1 type I collagen (*Col1A1*), α-1 type III collagen (*Col3A1*), tissue inhibitor of metalloproteinase 1, 2 and 3 (*TIMP1*, *2* and *3*) was performed using the QuantStudio 5 (Applied Biosystems, Waltham, MA, USA) and TaqMan Gene Expression Assays (Thermo Fisher Scientific, Waltham, MA, USA) (Supplemental Table S1). Relative quantification of gene expression was performed using the ΔΔCt method. Tata-box binding protein (*TBP*) was used as housekeeping gene.

### 2.4. Mass Spectrometry Imaging

#### 2.4.1. Sample Preparation

In order to facilitate sectioning, the aortas from day 7 were embedded in a mixture of 2% carboxymethyl cellulose (CMC, Sigma C4888, Waltham, MA, USA) and 4% gelatine (Sigma G1890: Gelatin from porcine skin, gel strength ~300 g Bloom, Type A) using 12 × 12 × 20 mm Peel-A-Way^®^ polyethylene embedding molds (Epredia, Kalamazoo, MI, USA). The contents of the molds were then frozen on a slurry of 2-propanol and dry ice for about 10 min until solid blocks were obtained and the molds were discarded. The tissue-containing blocks were stored at −80 °C before cryosectioning to a thickness of 10 µm. Tissue sections were thaw-mounted onto conductive indium tin oxide (ITO) glass slides (Bruker Daltonics, Bremen, Germany) and stored at −80 °C. Before matrix application for matrix-assisted laser desorption ionization mass spectrometry imaging (MALDI-MSI), mounted tissue sections were desiccated under vacuum for 20 min at room temperature.

The matrix solution was prepared by dissolving 60 mg of N-(1-Naphthyl)ethylenediamine dihydrochloride (NEDC, Sigma-Aldrich) in 6 mL of 70% methanol, premixed with 12 µL triethylamine (Sigma-Aldrich). Following 10 min of ultrasonification (Ultrasonic cleaner, VWR International, Radnor, PA, USA) an automated, pneumatic, TM-sprayer (HTX Technologies LLC, Chapel Hill, NC, USA) was used to spray the NEDC-matrix over the tissue sections. The matrix was sprayed in a criss-cross pattern for 14 passes at 80 °C and 100 µL/min (8 psi of N_2_) with 2 mm track spacing and a linear velocity of 1000 mm/min. Before analysis, the slides were scanned at 4800 dpi resolution using an Epson perfection V500 flatbed scanner (Seiko Epson Corporation, Nagano, Japan).

#### 2.4.2. MALDI-MSI Analysis

All MALDI–MSI experiments were performed using a timsTOF fleX dual-source MALDI mass spectrometer (Bruker Daltonics) at 10 µm spatial resolution. Prior to the MSI experiments, the slides underwent height correction and laser focus adjustment. Instrument calibration was performed in negative ion mode using clusters of red phosphorus in the *m*/*z* range of 70 to 1200 Da. During the runs, internal (i.e., lock mass) calibration was performed using the NEDC-matrix cluster ion (C_23_H_20_N_4_) at *m*/*z* 351.1615 [M-H]^−^. The laser power was optimized before each run and then held constant throughout the MALDI–MSI experiment. At each sampling position, 120 laser shots were used to acquire data and the tissue sections were analyzed in random order to prevent any possible bias arising from variations in the mass spectrometer sensitivity or matrix degradation over time.

#### 2.4.3. Data Processing

All MALDI-MSI data was analyzed using SCiLS Lab software (v.2023b Pro, Bruker Daltonics), with initial total ion count (TIC)-normalization. Following export of *m*/*z* intensity lists, untargeted statistical evaluation of the three different groups was performed using MetaboAnalyst v.6.0 software (https://www.metaboanalyst.ca/, accessed on 6 November 2025) [13]. After log_10_-transformation and autoscaling, evaluation was performed by one-way ANOVA, principal component analysis (PCA) and volcano plots (i.e., fold-change).

Peak *m*/*z* recalibration and preliminary metabolite identification were performed by using accurate mass measurements and the in-house developed software Met-ID (version 0.5.8) [14] which includes searchable metabolites entries from the Human Metabolome Database (HMDB) [15] and LIPID MAPS [16,17]. When possible, tandem MS (MS/MS)-fragmentation of candidate *m*/*z* features was performed to validate the metabolite identifications.

### 2.5. Statistical Analysis

Statistical analysis was performed with GraphPad Prism 10 (GraphPad software, San Diego, CA, USA). Data is reported as mean ± standard deviation. The Shapiro–Wilk test was used to determine normal distribution. Survival curves were compared with log-rank (Mantel–Cox) test, aortic diameter and gene expression results were compared by the Kruskal–Wallis test, followed by Dunn’s post hoc test. Disease incidence was compared by Fisher’s exact test. Aortic wall thickness and collagen/total area ratio were compared by one-way ANOVA, followed by Tukey’s post hoc test. Sample size was calculated to identify changes in aortic diameter of at least 50% between groups with a power of 80 and an α of 0.05.

## 3. Results

### 3.1. GLP-1 Receptor Is Expressed in Mouse Aortic Tissue

**Figure 2:**
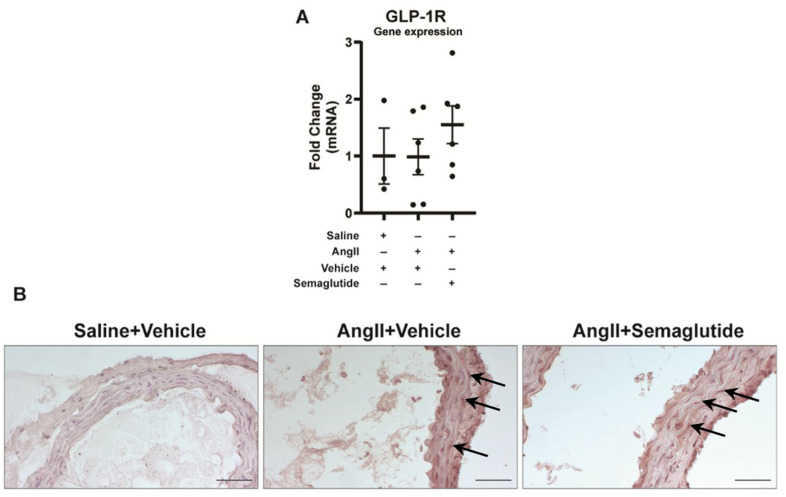
Detection of GLP-1 receptor in murine aorta. (**A**) Expression of *GLP-1R* mRNA in whole aortic tissue, on day seven. Analysis performed by the ΔΔCt method, TATA-binding box (*TBP*) used as housekeeping gene. Data expressed as fold change relative to the saline group (mean ± standard deviation). (**B**) Expression of GLP-1R in murine aortic tissue. Arrows indicate positively stained cells. Scale bar corresponds to 50 µm.

Expression of GLP-1R was verified at mRNA level in murine suprarenal aortic tissue on day seven. Its expression did not differ between non-induced controls, vehicle controls or semaglutide-treated mice (Figure 2A). Expression was also detected at tissue level, in the aortic wall, in the vehicle controls and semaglutide-treated groups (Figure 2B).

### 3.2. Semaglutide Prevents Aortic Rupture During the First Week of Disease Development

**Figure 3:**
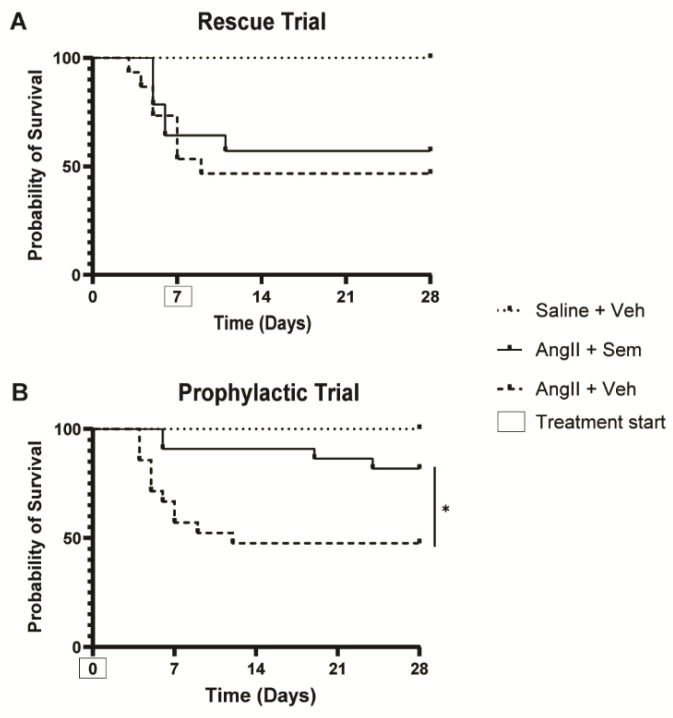
Probability of survival in (**A**) the rescue trial, with treatment starting on day seven of disease induction and (**B**) the prophylactic trial, with treatment starting simultaneously with disease induction. * Represents *p* < 0.05 angII + vehicle vs. angII + semaglutide. AngII: angiotensin II; Sem: semaglutide; Veh: vehicle.

In the rescue trial (before semaglutide was administrated), 7 out of 15 (46%) mice in the vehicle control group and 5 out of 14 (36%) in the semaglutide group died from an aortic rupture in the first seven days of disease induction by angII. After day seven, when semaglutide treatment started in one of the disease-induced groups, no differences in survival were observed between the vehicle and semaglutide groups (Figure 3A).

In the prophylactic trial, when semaglutide was administrated at the same day as disease induction, 9 out of 21 mice (43%) died in the vehicle control group from aortic rupture during the first seven days of disease induction. Strikingly, only 2 out of 22 mice (9%) died in the semaglutide-treated animals during the same period (Figure 3B). The probability of survival increased when treatment with semaglutide started simultaneously with disease induction (*p* < 0.05).

At the trial endpoint (28 days of disease induction with angII), average aortic diameter and AAA incidence did not differ between the vehicle control and semaglutide-treated groups, neither in the rescue trial nor in the prophylactic trials (Table 1). However, while the vehicle controls in the disease-induced mice developed a significant increase in the aortic diameter compared to the uninduced control animals, the semaglutide-treated mice did not reach a significant increase compared to the uninduced controls.

### 3.3. Semaglutide Promotes the Maintenance of Normal Aortic Wall Composition During the First Week of Disease Development

**Figure 4:**
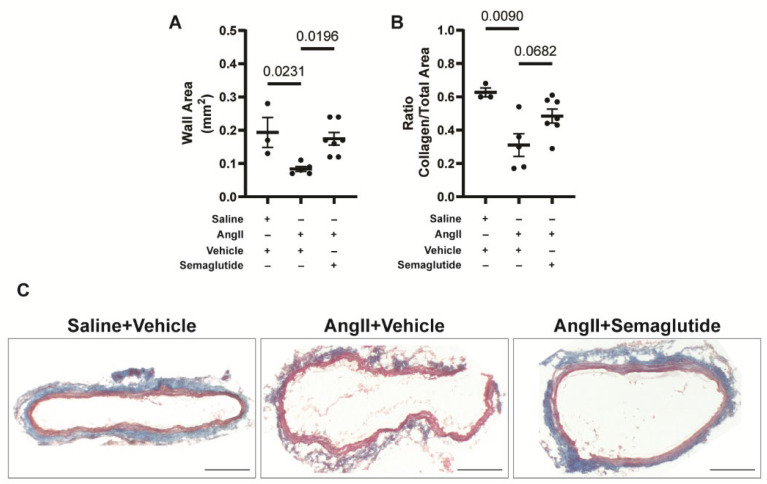
Histological analysis of the vessel wall. (**A**) Quantification of the total aortic wall area, by hematoxylin–eosin staining. (**B**) Quantification of the proportion of collagen to total aortic wall area, by Masson’s trichrome staining. (**C**) Representative images for Masson’s trichrome staining in the saline, vehicle and semaglutide groups. Collagen stained in blue, muscle in red, nuclei in black. Scale bar corresponds to 200 µm. Data are presented as mean ± standard deviation.

To understand how semaglutide prevents aortic rupture, aortic diameter and histological composition of the aortic wall were evaluated in an additional experiment after seven days of prophylactic treatment. At this time point, no mice had developed an aneurysm, but aortic diameter was increased in the vehicle control group administrated with angII (1.23 ± 0.08 mm), compared with vehicle controls not induced for disease (1.08 ± 0.03 mm, *p* < 0.05) and semaglutide-treated animals induced for disease with angII (1.08 ± 0.07 mm, *p* < 0.05) groups (Table 1). Histological analysis of the vessel wall with hematoxylin–eosin staining allowed to observe a reduced vessel wall thickness in the angII-induced vehicle-treated animals, compared to controls and semaglutide-treated angII-induced animals (Figure 4A). Analysis of the composition of the aortic wall by Masson’s trichrome staining allowed us to identify collagen (blue) and muscle (red). In the angII + vehicle group, the ratio of collagen area to total aortic wall area was reduced, compared to the saline + vehicle group. In the angII + semaglutide group, there was a trend for a higher proportion of collagen in the vessel wall, compared to the angII + vehicle group (see Figure 4B for quantification and Figure 4C for representative images). These results indicate that semaglutide prevented aortic rupture by day seven of disease induction, likely by maintaining an adequate proportion of collagen in the vessel wall.

The organization of collagen fibers in the ECM was evaluated by picrosirius red staining. Under polarized light, no differences were found in collagen fiber thickness and orientation between the groups after quantification (Supplemental Figure S1A). Elastin degradation was assessed by Verhoeff–Van Gienson staining. No differences were found between the groups (Supplemental Figure S1B).

### 3.4. Collagen Production and Degradation Balance at mRNA Level, During the First Week of Disease Development

To determine if the increased collagen content in the angII-infused semaglutide treatment group was a consequence of increased production or decreased degradation, mRNA expression of collagen 1 and 3, MMP 2 and 9 and TIMP 1, 2 and 3 were evaluated.

*Col1A1* and *Col3A1* mRNA expression was increased in the semaglutide-treated animals, and a trend for an increased expression in the vehicle control group was observed, compared to the non-induced controls. No differences in expression were found between vehicle controls and semaglutide-treated animals. *MMP2* mRNA was equally increased in the vehicle controls and semaglutide groups, compared to non-induced animals. Expression of *MMP9* mRNA did not differ between the three groups.

*TIMP1* mRNA expression was increased in the vehicle controls and semaglutide-treated groups, compared to non-induced animals, while *TIMP2* mRNA was only increased in the semaglutide-treated animals compared to non-induced animals, and *TIMP3* mRNA did not differ between groups (Table 2).

### 3.5. Potential Mechanisms as Determined by Mass Spectrometry Imaging Analysis

**Figure 5:**
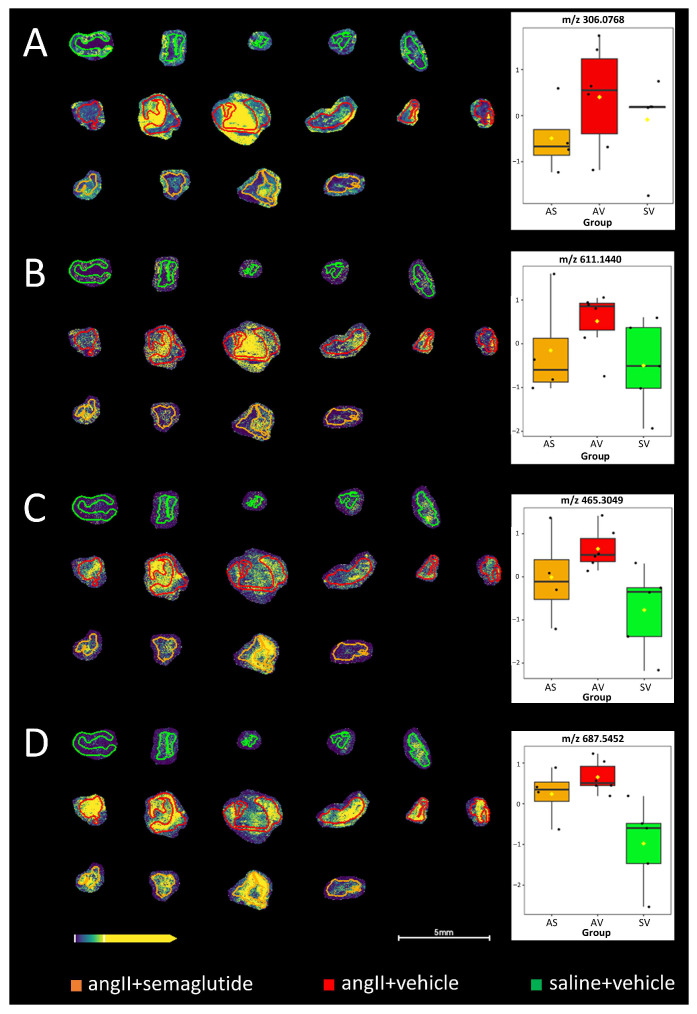
MALDI mass spectrometry imaging of the three experimental groups. (**A**) Glutathione (GSH) at *m*/*z* 306.0768 [M-H]^−^, obtained by met-ID and confirmed from MSMS-fragmentation (*p* = 0.032); (**B**) glutathione (GSSG) at *m*/*z* 611.1440 [M-H]^−^, identified based on accurate mass (*p* = 0.2443); (**C**) cholesterol sulphate at *m*/*z* 465.3049 [M-H]^−^ (*p* = 0.050); and (**D**) sphingomyelin (33:1) at *m*/*z* 687.5452 [M-H]^−^ (*p* = 0.009), obtained by met-ID, respectively. Coloration of the respective group regions is given at the bottom of the figure. AS: angII + semaglutide; AV: angII + vehicle; SV: saline + vehicle. Box-plots and *p*-values were obtained from one-way ANOVA of the three groups.

MALDI-MSI was used to identify potential novel mechanisms from the protective effects seen by semaglutide. Some interesting differences between the experimental groups were revealed by the untargeted analysis workflow (Figure 5). Even though the obtained differences for reduced and oxidized forms of glutathione (GSH and GSSG, respectively) were not significant (Figure 5A,B), increased levels were observed in the vehicle-control-treated group and there also appears to be a protective effect from the prophylactic semaglutide treatment, maintaining especially GSSG at a similar level as in the non-induced control group. Significant differences were obtained, however, between the groups for cholesterol sulphate and sphingomyelin (33:1) (Figure 5C,D).

**Figure 6:**
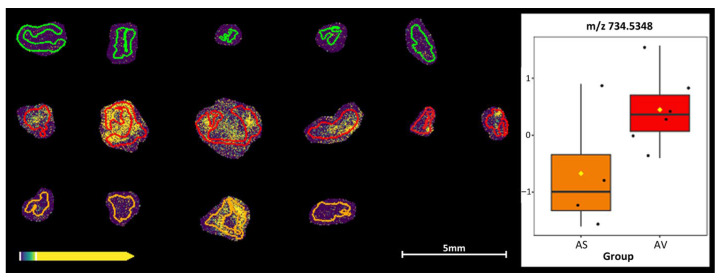
MALDI mass spectrometry imaging and box-plot of *m*/*z* 734.5348 (corresponding to the *m*/*z* of galactosylceramide (d18:1/16:0) [M+Cl]), obtained by met-ID, of the angII + semaglutide (orange) vs. the agII + vehicle (red) groups. The very low intensities in the saline + vehicle group (green) are also included in the figure for comparison.

A pairwise comparison between the semaglutide- and vehicle-control-treated groups was also performed, revealing a decreased intensity at *m*/*z* 734.5348 corresponding to galactosylceramide (d18:1/16:0) in the semaglutide (orange) group (Figure 6).

## 4. Discussion

GLP-1RAs can prevent AAA development in rodent models [18,19], and patients prescribed GLP-1RAs have significantly lower risks of mortality, AAA repair, and acute abdominal aortic syndrome [9]. In angII-infused Sprague Dawley rats, rescue treatment with lixisenatide prevents disease development through an increase in elastin content, a decreased expression of *MMP2* and *MMP9* and decreased infiltration of macrophages in the aortic wall [18]. In mice, prophylactic and rescue treatment with liraglutide, the first generation of GLP-1RAs, prevents AAA development in the angII and CaCl_2_ models, by preserving elastin content, decreasing *MMP2* and *MMP9* activity and reducing leukocyte infiltration [18,19].

In this study in the rescue trial, we observed that 41% of the mice infused with angII died from aortic rupture before the seven-day mark, and, thus, before the beginning of treatment. For the surviving mice, treatment with semaglutide did not affect AAA progression, as the average aortic diameter and incidence of disease at day 28 did not vary between vehicle and semaglutide groups. However, treatment with semaglutide did not increase the aortic diameter significantly higher than the control animals that were not induced for aneurysm with angII. This result could be affected by the relatively high mortality observed in this experiment, compared to the 20–30% that is anticipated, which could influence the effects in the surviving animals. In the prophylactic trial, survival was drastically improved in the semaglutide group. Nonetheless, at day 28, average aortic diameter and AAA incidence still did not differ between vehicle and semaglutide groups. Thus, semaglutide prevented aortic rupture in the early phases of disease development, but could not prevent AAA growth in the surviving mice as previous study with liraglutide.

To understand the effect of semaglutide during the first week of disease development, we conducted the seven-day prophylactic trial, where treatment started simultaneously with disease development, and was terminated after seven days. Aortic diameter at day seven was already increased in the vehicle-treated group, compared with non-induced controls and semaglutide-treated animals, confirming that disease had started to develop at this stage. At histological level, the aortic wall of the vehicle group was thinner than the non-induced controls and the semaglutide-treated groups. Increased aortic diameter and reduced aortic wall thickness are signals of a fragile vessel wall, explaining the propensity of this group to rupture. Semaglutide, by maintaining aortic wall thickness, reinforced the vessel and reduced the risk of dissection and rupture. Further analysis of the composition of the vessel wall identified a trend for a reduced proportion of collagen in the ECM of the vehicle control group, suggesting that the protective effect of semaglutide is associated with the maintenance of collagen in the aorta.

The collagen content of the aortic wall is regulated by the balance between synthesis and degradation. At mRNA expression level, *Col1A1* and *Col3A1*, the main forms of collagen present in the aorta, were increased in both the vehicle controls and semaglutide-treated groups. These groups also presented an upregulated expression of *MMP2*. Thus, the balance between production and degradation of collagen is similar in these groups, which does not explain the differences in collagen observed at tissue level. Inhibition of MMPs activity by TIMPs can affect this equilibrium. An increased expression of TIMPs indicates a reduced activity of MMPs. Our results show that expression of *TIMP2* is increased in the semaglutide-treated group, compared to non-induced controls. Hence, inhibition of *MMP2* activity is potentially higher in the semaglutide-treated animals, which indicates a reduced degradation of collagen compared to vehicle controls, and, thus, a higher proportion of collagen present in the aortic wall. Nonetheless, further experiments are necessary to determine the mechanism of action of semaglutide on collagen production and degradation using activity assays. Because all biopsies were used for histological analysis and RNA expression, no further material is available for this. Other mechanisms could also be responsible for this effect that was not identified in this study. One limitation of this study is that gene expression analysis and histology was only performed in animals that survived to the endpoint, which may create a bias because relevant information from the deceased mice is missing. Another possible explanation for the absence of effect on AAA inhibition could be due to the relatively low concentration of semaglutide administrated (12 µg/kg/day). This dose was chosen from the inhibitory effects seen on atherosclerosis by Rakipovski and colleagues [10]. We also tried a higher dose of 60 µg/kg/day but this did not affect the outcome. Additional limitations of this study include the sub-groups analysis, which includes few animals and may be underpowered.

The effect of semaglutide is dependent on the expression of the GLP-1R in the aorta. In mice, GLP-1R is detected in the aorta [20]. In humans, however, expression of GLP-1R in endothelial cells and VSMC is debated [21]. Nonetheless, GLP-1 and GLP-1RA have shown direct effects on vascular cells in vitro [21]. In our results, at mRNA level, GLP-1R is equally detected in all groups. Expression was confirmed at protein level, in the medial layer of the aorta, in vehicle controls and semaglutide-treated mice. Detection of the GLP-1R receptor in the aorta supports the hypothesis for direct effects of semaglutide in aortic rupture prevention.

MALDI-MSI was further used to identify potential novel mechanisms from the protective effects seen by semaglutide. Increased levels of putative reduced and oxidized forms of glutathione were observed in the vehicle-control-treated group and there also appears to be a protective effect from the prophylactic semaglutide treatment, maintaining especially oxidized forms of glutathione at a similar level to that in the non-induced control group. Significant differences were obtained between the groups for cholesterol sulphate and sphingomyelin (33:1). Ceramide and sphingomyelin are independent risk factors of atherosclerosis [22,23], and our previous results show a positive correlation of ceramide to T-cells and negative correlation to macrophages in perivascular adipose tissue in AAA [24]. A pairwise comparison between the semaglutide and vehicle-control-treated groups was also performed, revealing a decreased intensity at *m*/*z* 734.5348 in the semaglutide group. Using the met-ID software [14], the only obtained candidate metabolite at corresponding *m*/*z* 734.5348 is galactosylceramide (d18:1/16:0) [M+Cl]. Assuming that the identification is correct, this metabolite could, according to the literature, e.g., [25], reduce the development of angiotensin II-mediated aortic aneurysm. This could, in turn, potentially demonstrate the protective effect of prophylactic semaglutide administration. The causal interpretation of this metabolomic finding should be considered as a hypothesis-generating, rather than a confirmed, mechanism.

## 5. Conclusions

GLP-1RA agonist semaglutide prevents aortic dissection and rupture in the early phase of disease development in the angII mouse model, likely by promoting the maintenance of collagen in the aortic wall. These data suggest that GLP1-RAs might be considered in patients with a high dissection risk (Marfan and bicuspid valve disease).

## Figures and Tables

**Table 1:** Average aortic diameter aneurysm incidence and ruptures in the rescue and prophylactic trials, at day 7 or 28 of disease induction.

Treatment	Groups ¤	Aortic Diameter (mm)	Aneurysm Incidence §	Rupture Incidence
Rescue trial (28 days)	Saline (n = 4)	1.10 ± 0.12	0/4 (0%)	0/4 (0%)
	Vehicle (n = 7)	1.57 ± 0.43 *	3/7 (43%)	8/15 (53%)
	Semaglutide (n = 8)	1.40 ± 0.34	2/8 (25%)	6/14 (43%)
Prophylactic trial (28 days)	Saline (n = 6)	1.11 ± 0.08	0/6 (0%)	0/6 (0%)
	Vehicle (n = 10)	1.55 ± 0.39 *	6/10 (60%)	11/21 (52%)
	Semaglutide (n = 18)	1.36 ± 0.40	5/18 (28%)	4/22 (18%)
Prophylactic trial (7 days)	Saline (n = 4)	1.08 ± 0.03		
	Vehicle (n = 7)	1.23 ± 0.08 *#		
	Semaglutide (n = 8)	1.08 ± 0.07		

Data are presented as mean ± standard deviation. § incidence determined in animals that survived the trial and where samples were available. * Represents a *p*-value < 0.05, compared to saline in each trial. # Represents a *p*-value < 0.05, compared to semaglutide-treated animals. ¤ Represents number of animals that survived to ultrasound measurement.

**Table 2:** Gene expression (mRNA) of collagen, matrix metalloproteinases (MMPs) and tissue inhibitors of MMPs (TIMPs) in the aorta after seven days of disease induction.

	Saline + Vehicle (n = 4)	AngII + Vehicle (n = 6)	AngII + Semaglutide (n = 6)
*Col1A1*	1.00 ± 0.33	4.97 ± 2.44 *	6.53 ± 4.37 *
*Col3A1*	1.00 ± 0.24	3.81 ± 1.42 *	4.74 ± 3.04 *
*MMP2*	1.00 ± 1.00	3.13 ± 1.72 *	5.65 ± 4.68 *
*MMP9*	1.00 ± 1.00	0.81 ± 0.30	1.19 ± 0.82
*TIMP1*	1.00 ± 0.15	11.51 ± 5.10 *	9.99 ± 5.32 *
*TIMP2*	1.00 ± 0.13	1.57 ± 0.43	2.21 ± 0.85 *
*TIMP3*	1.00 ± 0.12	1.63 ± 0.55	1.40 ± 0.51

Analysis performed by the ΔΔCt method; TATA-binding box (TBP) used as housekeeping gene. Data presented as fold change relative to saline group (mean ± standard deviation). * Represents *p* < 0.05 vs. saline.

## Data Availability

The original contributions presented in this study are included in the article/Supplementary Material. Further inquiries can be directed to the corresponding author.

## Supplementary Materials

The following supporting information can be downloaded at: https://www.mdpi.com/article/10.3390/biomedicines14040933/s1, Supplemental Figure S1: Representative images for (A) Picrosirius red staining for collagen and (B) Verhoeff Van-Gieson staining for elastin, in the saline+vehicle, angII+vehicle and angII+semaglutide groups. Supplemental Table S1: TaqMan Gene Expression Assay ID.
