# Supplementary material for: Semaglutide Prevents Aortic Rupture and Dissection in the Angiotensin II Mouse Model
Source: Biomedicines. 2026 Apr 20;14(4):933. doi: 10.3390/biomedicines14040933 (PMC13113176; doi:10.3390/biomedicines14040933)
Supplement: Supplementary file 1 [file biomedicines-14-00933-s001.zip › biomedicines-4201419-SI.pdf]

Supplemental data

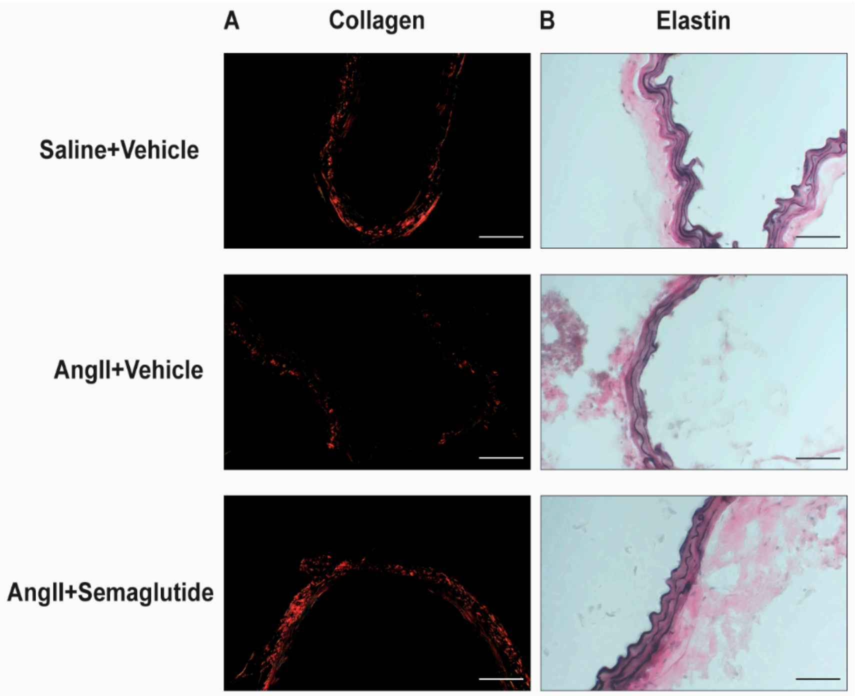

Supplemental Figure S1: Representative images for (**A**) Picrosirius red staining forcollagen and (**B**) Verhoeff Van-Gieson staining for elastin, in the saline+vehicle, angII+vehicle and angII+semaglutide groups.

Supplemental Table S1: TaqMan Gene Expression Assay ID.

Gene	TaqMan Assay ID
Col1A1	Mm00801666_g1
Col3A1	Mm01254476_m1
MMP2	Mm00439508_m1
MMP9	Mm00600163_m1
TIMP1	Mm01341361_m1
TIMP2	Mm00441825_m1
TIMP3	Mm00441826_m1
